# Willingness to Vaccinate (WTV) and Willingness to Pay (WTP) for Vaccination Against Peste des Petits Ruminants (PPR) in Mali

**DOI:** 10.3389/fvets.2019.00488

**Published:** 2020-01-15

**Authors:** Abdrahmane Wane, Michel Dione, Barbara Wieland, Karl M. Rich, Awa Sadio Yena, Abdou Fall

**Affiliations:** ^1^International Livestock Research Institute, Nairobi, Kenya; ^2^Centre de Coopération Internationale en Recherche Agronomique pour le Développement (CIRAD), Montpellier, France

**Keywords:** PPR, Mali, small ruminants, willingness to vaccinate (WTV), willingness to pay (WTP)

## Abstract

PPR remains a major challenge to smallholder farmers in Mali. To understand the drivers of low adoption of vaccination by farmers, we analyzed the socio-economic factors influencing farmer WTV during and in the absence of vaccination campaigns. Given that the costs associated with vaccination are largely borne by farmers, we assessed factors that associated with farmer willingness to pay (WTP) more than the current price (150 XOF per dose) by considering two attributes of improvement of the vaccines empirically highlighted as potential leverage points for intervention: access of farmers to vaccines (reducing the distance to the vaccine) and availability of information about the quality of the vaccine (introducing a vaccine viability detector). Data were collected in Mopti and Sikasso regions from 304 producers. Overall (*n* = 304), 89 percent of respondents vaccinated their herds during official vaccination campaigns. They are associated with receiving information on the campaign calendar more quickly if information is relayed at places of worship and if they have an awareness of the benefits of vaccination, including the protection of third parties. Only 39 percent of respondents vaccinate outside vaccination campaigns. They are positively linked to the credibility of private veterinarians and a recognition of the vital importance of vaccines but are negatively associated with ignorance of vaccination needs and concern about vaccine side-effects. Both distance-effects and quality-tracker effects are associated with farmer willingness to pay more than the current vaccine prices. Farmers practicing semi-intensive production systems are willing to pay 20 percent more than the current vaccine prices, as are users who believe in the beneficial effects of vaccination, users who consider the prices of vaccines as fair, and those who believe that some vaccines are more important than others. Factors that discourage producers from vaccinating or from paying more for vaccination would be more effectively managed with better communication on vaccine benefits through targeted information dissemination campaigns by Malian authorities. Greater price transparency throughout the vaccine production and deployment chain is critical, while timely availability of vaccine tested for viability would increase the willingness to vaccinate while improving access.

## Introduction and background

Livestock plays a critical role in Mali's economy. It represents 25% of the GDP of the primary sector and 11% of the national GDP. Livestock farming is the main source of income for over 30% of the population (1). At least 85% of rural households own domestic ruminants, with small ruminants (SR) representing a significant part of the livestock sector having ~40 million heads in 2016 (2). SR keeping provides readily available cash in the face of family needs, a source of livelihoods, medium-term assets, protein for daily meals, and socio-cultural functions. However, the multifunctional role of SR is threatened by the high burden of diseases, such as Peste des Petits Ruminants (PPR).

The principal method of control for PPR is vaccination which is reflected in the Global Control and Eradication Strategy for PPR^1^. There are many vaccines that are commercially available and have shown to be effective for at least 3 years post-vaccination (3, 4). However, most of them require the application of a strict cold chain during their deployment in the field. It is important that all SR are vaccinated because introduction of unvaccinated animals into a naïve population presents a high risk. Thus, the PPR Global Control and Eradication Strategy recommends at least 80% of vaccination coverage for SR above 3 months old (5).

Despite heavy investment of the public veterinary services of Mali in vaccination campaigns against PPR, countrywide vaccination coverage for SR is very low at just 7% (6). Nonetheless, demand exists for PPR vaccines, especially where innovative delivery mechanisms can be deployed. For instance, reports from ongoing development projects showed that up to 55% vaccination coverage in specific communes of the regions of Sikasso and Mopti (7) is possible using participatory approaches through Innovation Platforms to increase stakeholder participation in vaccination. However, there are many challenges encountered by stakeholders in the process of vaccination in Mali. First, private veterinarians still complain of unfair competition from State veterinarians in properly carrying out vaccination. Furthermore, vaccine delivery systems are often not very effective in reaching all SR livestock producers, particularly women, due to logistical problems caused by poor infrastructure, such as roads to reach remote villages and the absence of vaccination parks for SR. In addition, the cost of vaccination in Mali is largely borne by livestock farmers, constituting a barrier to participation given that not all livestock producers can afford it and some livestock producers do not feel there is enough benefit from investing in vaccination. Some stakeholders argue that the limited participation of livestock farmers in vaccination is not caused by the perceived high cost of vaccination, but rather poor access to good quality vaccines, together with a lack of awareness about timing of vaccination campaigns (6, 8). The maintenance of the cold chain throughout the vaccine delivery might also be a constraint.

The objective of this study was to assess farmer perceptions about vaccination of SR livestock, with particular emphasis on their willingness to vaccinate (WTV) and willingness to pay for vaccination (WTP). For the WTP, as already highlighted by Dione et al. (9) and Sadio (8), we considered the delivery of the vaccines at the closest area of residency (termed “distance-effect”) to facilitate accessibility and improved information on the quality of the vaccine (termed “quality tracker-effect” by introducing a vaccine viability detector^2^).

## Materials and Methods

### Literature Review and Theoretical Framework

Decisions for vaccination, whether human or livestock, can often be more associated with religious and spiritual reasons, personal opinions, safety worries and additional information, beyond any knowledge of risks, costs, and benefits (10, 11).

Through a qualitative study, Abakar et al. (11) identified a number of demand-side barriers to vaccination, including mistrust of vaccination programmes/services and health system issues, among mobile pastoralists in Chad. Given the singular relationships of Sub-Saharan pastoralists to their herds (12), it seems reasonable that vaccination hesitancy and refusal might be an issue for immunization operations against animal diseases. Once the decision to vaccinate is taken, and given vaccination is not free in Mali, it would be important to better understand the root causes or drivers of individual decisions to pay for vaccination services.

One approach to gaining such understanding is through the concept of *willingness to pay*, which is defined as the maximum price a consumer accepts to pay for a product or a service (13–16).

There are two main ways to measure the willingness to pay. The first approach, based on revealed preference and pioneered by Samuelson (17), holds that consumer preferences can be expressed through what they purchase under different incomes and prices. This perspective represents evidence-based choices from market data and various types of experiments (laboratory and field experiments or auctions). The second approach is based on stated preference which tries to determine the total economic value by incorporating both non-use value and option value through contingent valuation, conjoint analysis or contingent choice methods. Derived from direct and indirect surveys, the stated preference approach has been popularized by studies of the willingness to vaccinate or to pay for vaccines against human diseases (18–24) and recently against animal diseases (25).

**Figure 1:**
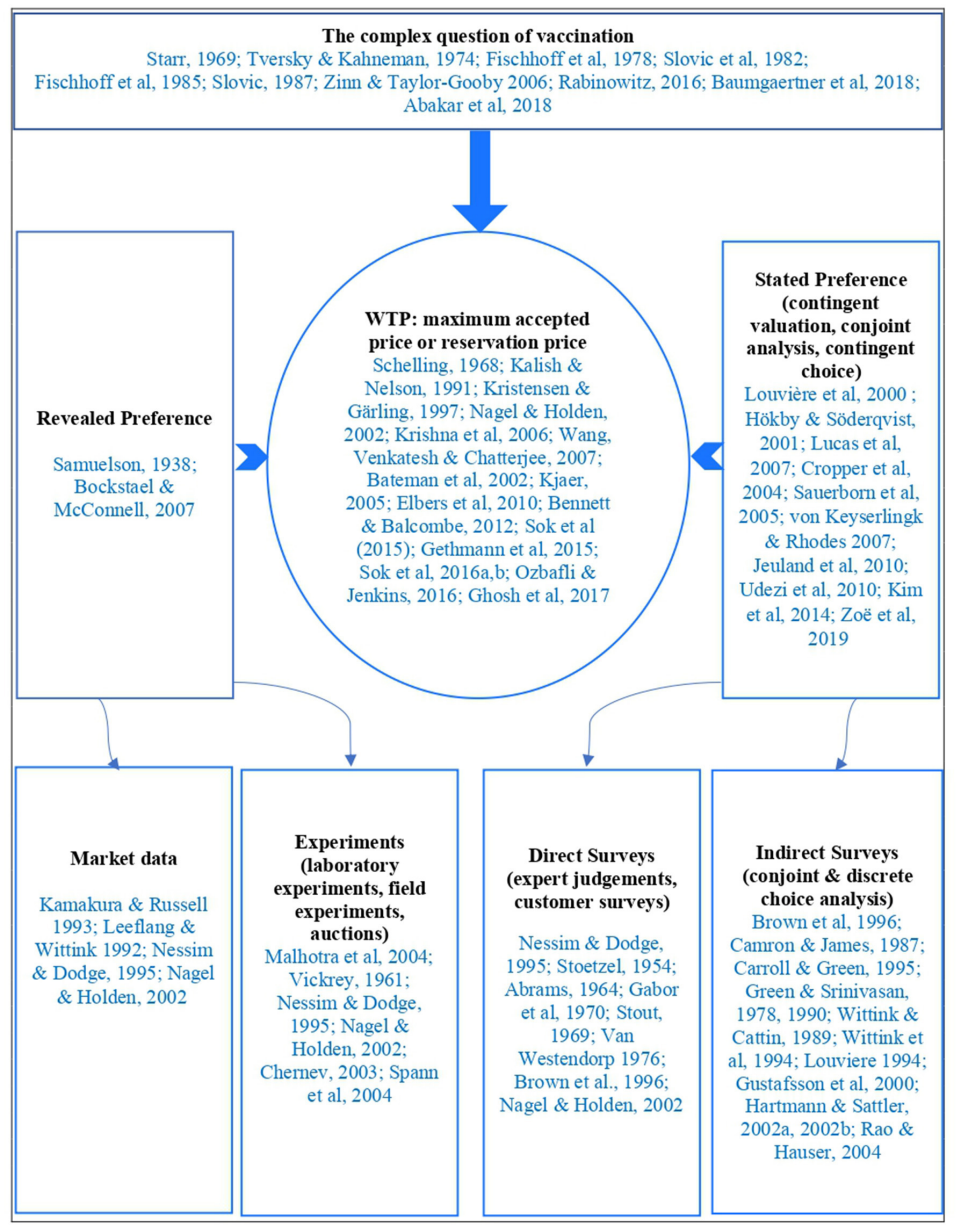
Vaccination behaviors and measurement methods of consumer's willingness to pay.

With regard to animal diseases, there is limited evidence describing the decision-making behind the vaccination of livestock. Elbers et al. (26) highlighted economic and social-psychological factors behind farmers' motivations to participate in a voluntary vaccination programme as well as their perceived need to actively be a part of the eradication campaign. Sok et al. (27–29) and Gethmann et al. (30) discussed the motivations, barriers, and willingness to vaccinate against bluetongue disease, while Bennett and Balcombe (31) investigated farmers' willingness to pay for a bovine tuberculosis (bTB) vaccine. These studies, however, focused on animal diseases in Europe. Their findings may be different to the situation of West African countries where vaccine coverage is low, health delivery systems insufficiently meet current needs, and effective communication approaches and tools are lacking (Figure 1).

### Methodology and Data

#### Objectives

Our overall objective is to assess the willingness of Malian livestock farmers to vaccinate (WTV) and their willingness to pay (WTP) for improved attributes of vaccines against PPR for SR. Two contingent concepts were analyzed separately, as some factors may affect WTV but not WTP for various reasons, such as farmer belief about the unfairness of vaccine pricing mechanisms. Therefore, we address two main questions:

- ***For the WTV model****: What are the socio-economic determinants of the attitude of livestock farmers regarding vaccination against PPR, i.e., in terms of choosing to vaccinate or not?*
- ***For the WTP model****: What are the socio-economic determinants associated with the farmer willingness to pay more than the current price for improved accessibility to the PPR vaccine (distance-effect) and quality of the vaccine (quality-tracker effect)?*^3^

#### Survey Tool

A questionnaire (see Appendix) was designed to collect data on household demographic characteristics, Production systems, vaccination knowledge and practices, constraints of livestock producers to vaccination and farmer WTV and WTP for vaccination, considering vaccine accessibility and quality.

#### Sample Size

**Map 1:**
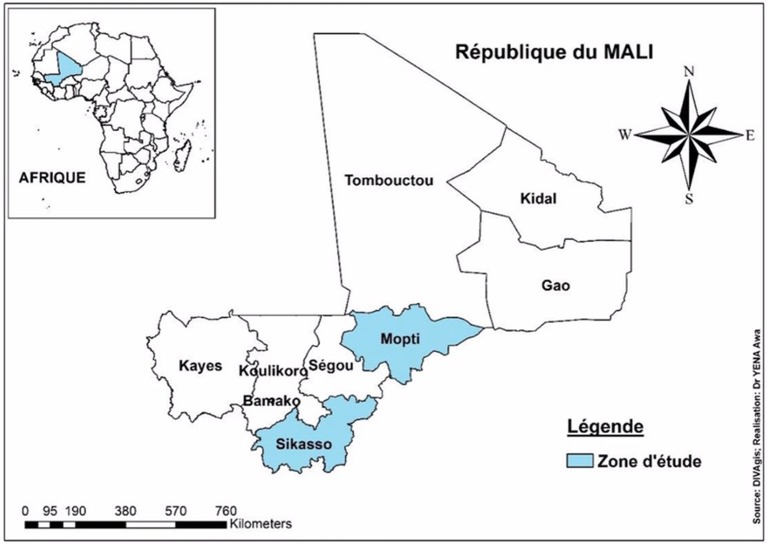
Localization of the study areas in Mali (in blue).

For our study, a sample was drawn from 4,254 producers who were identified as Feed the Future—Mali Livestock Technology Scaling (FTF-MLTS) program beneficiaries. We initially agreed to work with a margin of error of 3–5%, a confidence interval of 95% and a proportion of 50%. This involved selecting a sample size between 352 and 1,265 producers. Finally, due to access issues mainly related to insecurity^4^ and limited budget, 304 livestock farmers keeping either SR only or SR and cattle were reached. Among these livestock producers, 50% were from Mopti and 50% from Sikasso region (Map 1).

#### Data Collection and Processing

The survey tool was designed on ODK (Open Data Kit) and transferred to Samsung tablets for electronic capture. In each region, trained field veterinarians and veterinary technicians were recruited to administer the questionnaire to livestock producers. The team leader of the field activities oversaw data cleaning and quality assurance every day after the enumerators returned from the field. Data was then uploaded to the server and downloaded in Excel and statistical files for further cleaning and analysis.

The data were processed in several ways:

– *For willingness to vaccinate (WTV)*: Two binary variables were identified and used: (1) participation in vaccination campaigns and (2) use of vaccination outside of vaccination campaigns. To avoid overloading the analysis with a large number of variables, a correlation analysis was carried out to discriminate variables that have strong correlation with the identified binary variables.
– *For willingness to pay (WTP)*: Questions about WTP generated multiple responses which we considered as polychotomous dependent variables, requiring the use of a multinomial logistic regression.

Correlation and multicollinearity analysis allowed the identification of about fifteen independent variables for the estimation of WTV and WTP. Detailed explanations of the variables used in the regression analyses (described next) are presented in Table 1.

**Table 1:** Description of variables used in the regression analyses.

**Dependent variables**	**Independent variables** **(in order of appearance in the paper)**	**Modalities of independent variables** **(in order of appearance in the paper)**
**Willingness to vaccinate** **Yes = 1** No = 2	Information availability/access of/to vaccination campaign	Yes = 1 No = 2
	Information channel (multiple choices possible)	Radio = 1 Places of worship = 2 Town crier = 3 Word-of-mouth = 4
	Benefit of vaccination	Yes = 1 No = 2
**Willingness to pay for vaccination** No willingness to pay more = 0 Willingness to pay 5% more = 1 Willingness to pay 10% more = 2 Willingness to pay 20% more = 3	Cattle vaccination frequency	Every vaccination campaign = 1 Every year = 2 Many years = 3 Never = 4
	Vaccination protects others	Disagree = 1 Moderately agree = 2 Agree = 3
	Knowledge of vaccination needs	Yes = 1 No = 2
	Vaccination is vital for my animals	Disagree = 1 Moderately agree = 2 Agree = 3
	Private veterinarians (mandataries) are credible	Disagree = 1 Moderately agree = 2 Agree = 3
	Concerns about side-effects	Disagree = 1 Moderately agree = 2 Agree = 3
	Production system	Intensive production = 1 Semi-intensive production = 2 Extensive production = 3
	Animal species vaccinated	Cattle = 1 Sheep = 2 Goat = 3
	Fairness of PPR vaccine prices	Yes = 1 No = 2
	Some vaccines better than others	Yes = 1 No = 2
	Participation to vaccination campaign	Yes = 1 No = 2

#### Regression Analyses

We used a generic binary logistic regression analysis to better capture the socioeconomic factors that are associated with WTV and a multinomial logistic regression (Gologit model) to analyze WTP.

Binary logistic regression reflects situations in which the observed outcome for a dependent variable can have only two possible categories. Our study on WTV deals with “To vaccinate” vs. “Not to vaccinate.” For the multinomial logistic regression approach, its use represents situations in which the outcome can have three or more possible ordered or ranked responses. Our study on WTP involves multiple responses, such as “Not willing to pay a supplement,” or “Willing to pay a supplement of 5%,” “Willing to pay a supplement of 10%,” or “Willing to pay a supplement of 20%” for potential improvements in access to a vaccine and information on the quality of the vaccine.

We can generalize the Gologit model used in this paper by generalizing the bivariate logit model and considering an ordered dependent variable taking *j* modalities, written as:

(1) $${y_{i}}^{*}=\theta_{0}+\theta_{1}x_{1i}+\ldots +\theta_{k}x_{ki}+e_{i}=e_{i}\prime \theta +e_{i}$$

where *x*_1_…*x*_*k*_ are the regressors that influence y^*^, $y_{i}^{*}$ is latent, and *e*_*i*_ is the error term. As in the binomial case, the y^*^ modalities would depend directly on the position of y^*^ with respect to different threshold parameters or cutoffs that demarcate the boundaries of the various categories:

$$y=\{\begin{array}{c} 1\;if\;{y_{i}}^{*}<c_{1}\\ 2\;if\;c_{1}\leq \;{y_{i}}^{*}<c_{2}\\ .\\ .\\ .\\ J\;if\;y_{i}^{*}>c_{J-1} \end{array}$$

By defining *F* as the function for distributing error terms that follows a logistic law, we have:

(2) $$Prob\left(y_{i}=1\right)=Prob\left(x_{i}\prime \theta +e_{i}<c_{1}\right)=F\left(c_{1}-x_{i}\prime \theta \right)$$

(3) $$\begin{array}{l} Prob\left(y_{i}=j\right)=Prob\left(c_{j-1}\leq x_{i}\prime \theta +e_{i}<c_{j}\right)\\ \;=F\left(c_{j}x_{i}\prime \theta \right)-F\left(c_{j-1}-x_{i}\prime \theta \right),\;2\leq J\leq J-1 \end{array}$$

(4) $$\begin{array}{l} Prob\left(y_{i}=J\right)=Prob\left(x_{i}\prime \theta +e_{i}>c_{j}\right)\\ \;=1-F\left(c_{j-1}-x_{i}\prime \theta \right) \end{array}$$

The model coefficients θ are estimated by maximum likelihood. In addition, it is essential to understand and test an implicit hypothesis of this model, known as the parallel regression hypothesis, for the ordered logit model, and the odds proportion hypothesis (32–34).

Equations (2–4) can be used to derive the cumulative probabilities that are written in the simplified form by: $Prob\left(y_{i}\leq j\right)=F\left(c_{j}-x_{i}\prime \theta \right),1\leq j\leq J-1$.

These last equations show that the ordered regression model is equivalent to *J* − 1 binary regressions under the fundamental assumption that estimated coefficients with respect to the explanatory variables are identical in each of the equations.

In contrast to binary models, interpreting the coefficients of an ordered model is complicated, especially for intermediate modalities. To do so, we calculate the marginal effects of variables on the probabilities (as in Equations 5–7) and resort to the transformation of coefficients into odds ratios or conditional probabilities.

(5) $$\begin{array}{l} \frac{\partial Prob\left(y_{i}=1|\;x_{i}\right)}{\partial x_{ik}} \end{array}$$

(6) $$\begin{array}{l} \frac{\partial Prob\left(y_{i}=j|\;x_{i}\right)}{\partial x_{ik}} \end{array}$$

(7) $$\begin{array}{l} \frac{\partial Prob\left(y_{i}=J|\;x_{i}\right)}{\partial x_{ik}} \end{array}$$

There are two ways to determine the overall marginal effects on the sample: evaluate at mean data value or evaluate for each observation and calculate the average of the individual marginal effects in the sample. For large samples, both methods give similar results. For our paper, we chose to calculate the marginal effects in relation to the median individual.

When outcome variables are ordinal, the ordinal logit model has been popularly used. However, some researchers, such as Williams (33, 34) prefer to use the Generalized ordered logit/partial proportional odds models (Gologit/ppo) as they provide more robust results even if the interpretation of model outcomes becomes more difficult. Therefore, Williams (33, 34) writes the Gologit model as:

(8) $$\begin{array}{l} P\left(Y_{i}>j\right)=\frac{\exp\left(\theta_{i}+X_{i}\beta_{j}\right)}{1+\left[\exp\left(\theta_{i}+X_{i}\beta_{j}\right)\right]},\;with\;j=1,2,\ldots ,M \end{array}$$

where M is the number of categories of the ordinal dependent variable.

Finally, the probabilities that *Y* will take on each of the values 1, 2, …, *M* can be determinated by:

(9) $$\begin{array}{l} P\left(Y_{i}=1\right)=1-g\left(X_{i}\beta_{1}\right) \end{array}$$

(10) $$\begin{array}{l} P\left(Y_{i}=j\right)=g\left(X_{i}\beta_{j-1}\right)-g\left(X_{i}\beta_{j}\right)\;with\;j=2,\ldots ,M-1 \end{array}$$

(11) $$\begin{array}{l} P\left(Y_{i}=M\right)=g\left(X_{i}\beta_{M-1}\right) \end{array}$$

Depending on the values of M, it would be possible to have an equivalent of a logistic regression model (*M* = 2), or a series of binary logistic regressions (*M* > 2).

#### Context and Study Area

Livestock vaccination is run through public-private partnership. It is mainly carried out by established private veterinarians called “*mandataires*” (or mandataries) under the supervision of the public veterinary services except in areas where these public veterinary services are not established. In high insecurity regions, vaccination is provided free of charge by the government or some development organizations. In contrast, private veterinarians fully recover the cost of vaccination from farmers. Every year, official vaccination campaigns for livestock are launched by the Government in early October and will last to March. However, given random sources of funding and mobility of livestock keepers, farmers who miss this vaccination campaigns can get their animals vaccinated by available veterinarians in their communities at any time of the year; this is referred to as “outside vaccination campaigns.”

The FTF-MLTS program seeks to contribute to the inclusive growth of the ruminant livestock value chains for increased income, food and nutrition security for 266,000 cattle, sheep, and goat keepers and other value chains actors in three regions in the country (Mopti, Timbuktu and Sikasso), as a means of lifting them out of poverty. Supported by the United States Agency for International Development (USAID) as part of the US government's Feed the Future initiative, the program sets out to bridge ruminant livestock productivity gaps and to enhance the volume and value of ruminant livestock marketed through a wide-scale dissemination of proven livestock technologies and best practices. The FTF-MLTS program has made priority investments in designing and rolling out innovative approaches to increase vaccination coverage of SR and cattle against PPR and Contagious Bovine Pleuro-Pneumonia (CBPP), respectively and bovine/ovine pasteurellosis (7).

## Results

Contingent valuation methods for eliciting preferences for non-marketed goods are useful in addressing actor WTP. In this survey, we first asked farmers if they are willing to pay 20% more than the current PPR vaccine price, then 10% more and finally 5% more if the health services were delivered at the closest area of residency (distance-effect) to facilitate accessibility. The same questions were asked for improved information about the quality of the vaccine (quality-tracker effect).

Almost all livestock producers (96%) perceive tangible benefits of vaccines for herd size, as they expect fewer animal losses. However, while 44% of them are not aware that vaccinating their herds can also protect those of others, 29% thought that vaccination is required only during outbreaks, and 24% believed that vaccination serves to fatten animals.

### Regression Analysis Results for the Willingness to Vaccinate (WTV)

Almost 89% of the 304 respondents vaccinate their herds during the vaccination campaigns formally organized by public authorities while 11% of them did not vaccinate. Outside formal vaccination campaigns, only 39% of respondents vaccinate their herds while 61% stated that they did not vaccinate outside the period of organized campaigns (Table 2).

**Table 2:** Distribution of farmers' responses on vaccination participation.

**Variable**	**Modalities**	**Numbers**	**%**
Vaccination campaign	Yes: 1	272	89.474
	No: 2	32	10.526
Outside the vaccination campaign	Yes: 1	119	39.145
	No: 2	185	60.855

#### Factors Associated With the WTV During Vaccination Campaigns

WTV during vaccination campaigns was found to be significantly associated with the availability, access and attributes of information provided about the vaccination campaigns (Table 3).

**Table 3:** Factors associated with the WTV during vaccination campaigns.

**Logistic regression**			**LR chi^**2**^(10) = 155.38**			
**Number of observations = 304**			**Log likelihood = −24.606224**			
**LR chi2(10) = 155.38**			**Pseudo ***R***^**2**^ = 0.7595**			
**Variables**		**Coefficient**	**Odds Ratio**	**95% confidence interval**		***P*****-value**
Information availability/access	Yes^*^					
	No	−4.8	0.008	0.0008	0.0874	<0.001
Information channel	Radio^*^					
	Places of worship	2.7	0.070	0.0043	1.1342	0.061
	Town crier	1.0	0.364	0.0277	4.7699	0.441
	Word-of-mouth	0.7	0.506	0.0513	4.9889	0.560
Benefit of vaccination	Yes^*^					
	No	−2.3	0.102	0.0147	0.7134	0.021
Cattle vaccination frequency	Every vaccination campaign^*^					
	Every year	−4.1	0.016	0.0012	0.2357	0.002
	Many years	−5.7	0.003	0.00005	0.2184	0.007
	Never	−4.3	0.014	0.0006	0.3557	0.010
Vaccination protects others	Disagree^*^					
	Moderately agree	0.8	2.279	0.1125	46.1685	0.591
	Agree	4.9	129.431	1.1750	14257.18^**^	0.043

*A common practice would consist to fuse the reference category with the other levels of the variable that are not significantly different from the reference category. We proceed to these supplemental analyses but this process did not give conclusive results. For the “Information channel” variable, the new reference category resulted from the regrouping provided p-values of 0.868 and 0.771 for the odds ratios*.

* *Reference category*.

** *Abnormally wide confidence interval can raise with small sample size or when some variables have several categories with small frequencies*.

Given the prominent place of religions in Mali, places of worship play an important role for information sharing. Through them, information on vaccination campaigns is more effective in incentivizing actors to vaccinate their animals. Previous experiences with cattle vaccination could, however, constraint the care of small ruminants as this factor is negatively associated with the WTV. For an equivalent price per dose of vaccine, there seems to be a trade-off between the different species to protect. The respective odds ratio of each of these attributes, however, is relatively small, implying a limited effect on the probability of participating in vaccination campaigns.

In Malian rural areas, scrutiny and judgment of community members are important social values that reinforce peer effects. Recognition that vaccinating can help to protect herds other than those owned by themselves constitutes an important incentive for vaccination. With regard to the potential impacts on third parties, the farmers who agree that vaccination protects their herds have a WTV that is 129.4 times more often than the farmers who disagree.

#### Factors Associated With the WTV Outside Vaccination Campaigns

Even though only 39% of respondents claim to vaccinate their flocks outside of official vaccination campaigns, we observe that lack of knowledge about vaccination needs (different from vaccination benefits) and concerns about side-effects discourage actors from vaccinating (Table 4). This may be due to the presence of less experienced or non-trained technicians handling vaccination outside of official campaigns, leading to a greater incidence of side effects due to poor vaccination techniques. On the other hand, the credibility of private veterinarians (referred to as the variable “Mandataries are credible”) and the recognition of the vital importance of the vaccines were all shown to have a positive effect on their WTV. The strong odds ratios indicate that farmers who moderately and fully agree that vaccination is vital have a WTV that is 243 to 262 time higher compared to farmers who disagree (Table 4).

**Table 4:** Factors influencing the WTV outside the vaccination campaigns.

**Logistic regression**						
**Number of observations = 304**			**Prob > chi^**2**^ = 0.000**			
**LR chi^**2**^-11 = 78.37**			**Pseudo ***R***^**2**^ = 0.1926**			
**Variables**		**Coefficient**	**Odds ratio**	**95% confidence interval**		***P*****-value**
Information channel	Radio^*^					
	Places of worship	0.1	0.9	0.2	3.9	0.936
	Town crier	1.1	0.3	0.1	0.9	0.038
	Word-of-mouth	0.9	0.4	0.1	1.2	0.106
Knowledge of vaccination needs	Yes^*^					
	No	−1.0	0.4	0.2	0.8	0.006
Vaccination is vital for my animals	Disagree^*^					
	Moderately agree	5.5	243.3	10.8	5,477.8^**^	0.001
	Agree	5.6	261.8	14.6	4,689.0^**^	<0.001
Private veterinarians (mandataries) are credible	Disagree^*^					
	Moderately agree	1.3	0.3	0.1	1.2	0.080
	Agree	2.2	0.1	0.0	0.4	0.002
Concerns about side-effects	Disagree^*^					
	Moderately agree	−2.3	0.1	0.0	0.3	<0.001
	Agree	−1.2	0.3	0.1	0.7	0.004

* *Reference category*.

** *Abnormally wide confidence interval can raise with small sample size or when some variables have several categories with small frequencies*.

### Regression Analysis Results for Willingness to Pay (WTP) for Vaccination

We recoded the variables and created new ones to allow their use in an ordered logit regression: ***Distv***and **Qv** were the variables measuring the willingness to pay a premium for vaccine delivery to be significantly shortened and for a quality-tracker to be implemented on the vaccine packages, respectively. Their modalities are: “1” if the farmer is willing to pay 5% more on the current price of the vaccine; “2” if he/she is willing to pay 10% more; “3” if he/she is willing to pay 20% more and “0” if he/she refuses all three options and therefore does not want to pay anything more on the price of the vaccine. From field investigations, a large majority of farmers (69% and 71%) say they are willing to pay 20% more than the current price of the dose of PPR vaccine if, respectively, the delivery distance and quality-tracking of the vaccines are improved (Table 5). It should be noted that between 13 and 14% of farmers say they are not prepared to pay more regardless of the improvement made in the delivery and quality-tracking of vaccines, respectively.

**Table 5:** Distribution of dependent variables on the WTP for distance and quality parameters.

**Distv**	**Freq**.	**Percent**	**Cum**.	**Qv**	**Freq**.	**Percent**	**Cum**.
0	43	14.14	14.14	0	39	12.83	12.83
1	11	3.62	17.76	1	9	2.96	15.79
2	40	13.16	30.92	2	38	12.50	28.29
3	210	69.08	100.00	3	218	71.71	100.00
Total	304	100.00		Total	304	100.00	

A generalized ordered logit (Gologit) model was used to address shortcomings of the ordered logit model and parallel-lines model as stated by the Brant's test (33, 34), which rejected the parallel regression assumption (Table 6).

**Table 6:** Test of parallel regression assumption.

	**Chi^**2**^**	***df***	***p* > Chi^**2**^**
Brant	44.21	14	0.000

Based on the existing literature, our knowledge of the Malian context, and the use of a stepwise approach, the following predictive variables were included:

– *For the distance-effect*-***Distv***: “Production system,” “Animal species vaccinated,” “Mandataries are credible,” “Fairness of PPR vaccine prices,” “Benefit awareness,” “Some vaccines better than others,” “Cattle vaccination frequency.”
– *For the quality-tracker effect*-***Qv***: “Production system,” “Animal species vaccinated,” “Mandataries are credible,” “Fairness of PPR vaccine prices,” “Benefit awareness,” “Some vaccines better than others,” “Cattle vaccination frequency,” “Vaccination campaign participation.”

Finally, the regression was done successively on the distance-effect (**Distv**) and the quality-effect (**Qv**).

#### For the Distance-Effect: Distv

Table 7 shows that, all other things being equal, farmers in semi-intensive production systems, those who perceive that PPR vaccine prices are fair, that vaccination is beneficial, including the comparative advantage of PPR vaccines, are willing to pay a premium if the physical access of vaccines is improved.

**Table 7:** Regression analysis results for the distance-effect.

**Dependent variable: Distance-Effect (WTP 0%, 5%, 10%, 20)**		**Generalized ordered logit model**					
		**Distv** **=** **0**		**Distv** **=** **1**		**Distv** **=** **2**	
**Independent variables**		**Coefficient**	**t-statistic**	**Coefficient**	**t-statistic**	**Coefficient**	**t-statistic**
Livestock production system	Extensive^*^						
	Semi-intensive	−0.868	0.010	−0.868	0.010	−0.868	0.010
	Intensive	0.074	0.952	0.073	0.952	0.074	0.952
Benefit of vaccination	Yes^*^						
	No	−1.925	0.007	−1.925	0.007	−1.925	0.007
Fairness of PPR vaccine prices	Yes^*^						
	No	−2.733	0.000	−2.151	0.000	−1.367	0.000
Some vaccines better than others	Yes^*^						
	No	−0.465	0.263	−1.059	0.003	−1.178	0.000
Constant		3.576	0.000	3.474	0.000	2.514	0.000

* *Reference category*.

The regression analysis further reveals that the coefficients for “Production system” and “Benefit awareness” do not vary across the categories of the response variable, i.e., the distance-effect. This means “Production system” and “Benefit awareness” have positive impacts on the distance-effect. Therefore, the more the farmers in semi-intensive production system are aware of the benefits of vaccination, the greater their willingness to pay a premium for a shorter vaccine delivery distance.

These trends are visible only through sign and significance at this stage. To improve the quality of interpretation of the regression results (Table 8), we tabulate the marginal effects and coefficients into odds ratios or conditional probabilities.

**Table 8:** Marginal effects of the variables used in the model on distance parameter.

	**Marginal effects**	**t-statistics**	**95% confidence interval**	
**Fairness of PPR vaccine prices**				
Pr(Distv = 0); independent variable = 1	0.076	0.000	0.043	0.109
Pr(Distv = 1); independent variable = 1	0.045	0.001	0.019	0.071
Pr(Distv = 2); independent variable = 1	0.144	0.000	0.102	0.186
Pr(Distv = 3); independent variable = 1	0.735	0.000	0.683	0.786
**Benefit awareness**				
Pr(Distv = 0); independent variable = 1	0.127	0.000	0.093	0.162
Pr(Distv = 1); independent variable = 1	0.034	0.001	0.014	0.053
Pr(Distv = 2); independent variable = 1	0.131	0.000	0.093	0.168
Pr(Distv = 3); independent variable = 1	0.708	0.000	0.658	0.758
**Production system**				
Pr(Distv = 0); independent variable = 2	0.167	0.000	0.126	0.208
Pr(Distv = 1); independent variable = 2	0.039	0.000	0.017	0.061
Pr(Distv = 2); independent variable = 2	0.143	0.000	0.102	0.184
Pr(Distv = 3); independent variable = 2	0.650	0.000	0.592	0.709
**Some vaccines better than others**				
Pr(Distv = 0); independent variable = 2	0.155	0.000	0.109	0.202
Pr(Distv = 1); independent variable = 2	0.064	0.001	0.028	0.101
Pr(Distv = 2); independent variable = 2	0.172	0.000	0.117	0.226
Pr(Distv = 3); independent variable = 2	0.608	0.000	0.540	0.677

Farmers declare that they are willing to pay a higher price than the current vaccine price if physical access to it is improved by a significant reduction in the distance of supply and also if other conditions are met. When farmers consider the price of PPR vaccines to be fair, their probability of paying 20% more than the current price of a vaccine dose increases by 73%. When they are well aware of the beneficial effects of vaccination against PPR, the probability increases by 71%. When farmers are in semi-intensive production system, the probability of paying 20% more increases by 65%. When they believe that some vaccines are better than others, the probability increases by 61%.

#### For the Quality-Tracker Effect: Qv

WTP for improved quality tracking of PPR vaccines is associated with the same significant variables: “Production system,” “Benefit awareness of PPR vaccination,” “Fairness of the PPR vaccine prices,” and “Comparative advantages of some vaccines” (Table 9). When respondents indicate “No” to one or more of these variables, this has a negative impact on WTP for a vaccine-quality tracker. Thus, when a farmer is frustrated about these variables (e.g., feels prices are not fair), it reduces their willingness to pay a premium above the current vaccine prices (Table 9).

**Table 9:** Regression analysis results for the quality-effect.

**Dependent variable: Quality-Effect (WTP 0%, 5%, 10%, 20)**		**Generalized ordered logit model**					
**Independent variables**		**Qv** **=** **0**		**Qv** **=** **1**		**Qv** **=** **2**	
		**Coeff**.	**t-statistic**	**Coeff**.	**t-statistic**	**Coeff**.	**t-statistic**
Fairness of PPR vaccine prices	Yes^*^						
	No	−2.911	0.000	−2.592	0.000	−1.565	0.000
Benefit of vaccination	Yes^*^						
	No	−1.722	0.019	−1.722	0.019	−1.722	0.019
Some vaccines better than others	Yes^*^						
	No	−0.956	0.001	−0.956	0.001	−0.956	0.001
Participation to vaccination campaign	Yes^*^						
	No	0.528	0.336	0.528	0.336	0.528	0.336
Livestock production system	Extensive^*^						
	Semi-intensive	−0.838	0.026	−0.838	0.026	−0.838	0.026
	Intensive	−0.214	0.858	−0.214	0.858	−0.214	0.858
Constant		4.018	0.000	3.592	0.000	2.458	0.000

* *Reference category*.

If they are convinced that vaccine prices are fair, their probability of paying 20% more on the current price of vaccines increases by 77% (Table 10). In the same way, if they are aware of the benefit of PPR vaccination, the probability for paying the vaccines 20% more is 73% (Table 10). The practice of semi-intensive production activities also leads them to an increase in the probability of paying 20% more to 68% if the quality of vaccines is improved. And finally, the awareness of farmers about the relative comparative advantage of some vaccines increases their probability of paying 20% more for vaccines than their current prices by 82% (Table 10).

**Table 10:** Marginal effects of the variables used in the model on quality parameter.

	**Marginal effects**	**t-statistics**	**95% Confidence Interval**	
**Fairness of the PPR vaccine prices**				
Pr(qv = 0), Independent variable = 1	0.065	0.000	0.036	0.095
Pr(qv = 1), Independent variable = 1	0.030	0.004	0.010	0.050
Pr(qv = 2), Independent variable = 1	0.139	0.000	0.010	0.181
Pr(qv = 3), Independent variable = 1	0.765	0.000	0.715	0.815
**Benefit awareness**				
Pr(qv = 0), Independent variable = 1	0.117	0.000	0.084	0.150
Pr(qv = 1), Independent variable = 1	0.029	0.002	0.010	0.047
Pr(qv = 2), Independent variable = 1	0.122	0.000	0.086	0.158
Pr(qv = 3), Independent variable = 1	0.733	0.000	0.684	0.781
**Production system**				
Pr(qv = 0), Independent variable = 2	0.151	0.000	0.110	0.192
Pr(qv = 1), Independent variable = 2	0.033	0.002	0.012	0.054
Pr(qv = 2), Independent variable = 2	0.137	0.000	0.096	0.178
Pr(qv = 3), Independent variable = 2	0.679	0.000	0.619	0.739
**Some vaccines more important than others**				
Pr(qv = 0), Independent variable = 1	0.081	0.000	0.046	0.116
Pr(qv = 1), Independent variable = 1	0.018	0.008	0.005	0.031
Pr(qv = 2), Independent variable = 1	0.083	0.000	0.047	0.119
Pr(qv = 3), Independent variable = 1	0.818	0.000	0.751	0.885

## Discussion

PPR is a concern for the Malian livestock sector where the vaccination coverage is still very low. Although effective vaccines are available, the disease remains endemic for various reasons. Many of these reasons relate to the willingness of livestock producers to vaccinate or to pay for vaccination. Our study focused on socioeconomic factors influencing the WTV and WTP for vaccines against PPR in Sikasso and Mopti regions in Mali. The study led to interesting findings which highlight a number of important policy implications.

First, the place and function of beliefs in the decision-making process are often neglected, even though they can validly be the result of rational behavior. Vaccination programmes against animal diseases are mostly evaluated by using availability and access to vaccines. Although these two elements are very important, it appears that farmer beliefs associated with their participation in vaccination programmes are crucial to understand their decision-making process to vaccinate or pay for vaccination (26–30). The challenge of considering farmer beliefs and perceptions about vaccination is to better understand their behavior, but also to develop appropriate policy instruments to increase their participation in vaccination campaigns and improve the effectiveness and efficiency of voluntary vaccination strategies (26). Our study places greater emphasis on the behavior of farmers and shows that their decisions are based on their perceptions of the characteristics and effects of vaccination against PPR.

Second, market orientation (semi-intensive production) plays an important role in the willingness to pay for vaccination. The use of fattening operations, while still maintaining some flexibility for animal mobility, suggests a stronger market orientation compared to the extensive system. Vaccination, even when paid for, appears to be a part of this strategy, more than in other production systems.

Third, our results show that making information on vaccination campaigns more accessible and livestock producers more aware of the benefits of vaccines, may change their WTV and WTP. This requires working both on the content and form of information dissemination. The information must go beyond simply informing livestock producers about the dates and periods of vaccination campaigns. Rather, such information needs to effectively communicate the positive role of vaccines in the control of animal diseases; on their protective effects on the herd and those of neighbors; and their potential side effects, whether positive or negative.

Dissemination of information through different media, such as places of worship, communal radio stations, mouth-to-mouth, etc. have proven to be somewhat effective, though future research must accurately assess these platforms in greater depth. All these media require physical access while there are great opportunities to expand information access through innovative ways with the growing accessibility of internet-based web applications and mobile phones in this country.

Fourth, the results highlight the negative impact of livestock farmers' perceptions about inequity of vaccine prices. Our study does not directly show this; however, discussions we had after the study indicate that farmers seem to perceive differentiated (and unfair) vaccine pricing between cattle and SR that does not consider the differences in size and value of each species. This involves trade-off behaviors between vaccinating cattle and small ruminants. They do not seem to be aware of the costs of producing and deploying the vaccines to be considered. Therefore, the Malian authorities might benefit from including greater price transparency throughout the vaccine production and deployment chain by facilitating an equal access to and greater clarity about price information. For instance, enhanced communication about the subsidies supported by Malian authorities could help in this transparency.

Fifth, regarding farmers' trust of private veterinarians, the FTF-MLTS program took an important step to address this lack of trust through better planning of vaccination campaigns using participatory approaches vaccination delivery through Innovation Platforms (IPs). Preliminary results show that IPs have been able to strengthen trust between farmers and private veterinarians, consequently improving performance of vaccination campaigns (9).

Finally, results of our model clearly suggest that distance and quality perceptions are critical issues underpinning farmer willingness to pay for improved vaccines. Developing logistical support and efficient supply chains that improve access to vaccines in a timely manner is critical. Porphyre et al. (35) showed that the initial availability of vaccine stock at the start of an outbreak significantly contributes to optimal control strategies for disease outbreaks. Therefore, development of basic infrastructure and control of the cold chain are critical in the implementation of optimal health delivery systems. This can explain the respondents' desire and WTP for a test that shows them whether a vaccine is viable.

The main limitation of this study is intrinsically linked to the inaccessibility of some areas due to the security problems that have characterized the country for more than a decade. This security situation is even more tense in the livestock areas in particular from Mopti region to the extreme Northern part of the country. Thus, the sampling of households to be surveyed was carried out only in accessible areas, particularly those targeted by the FTF-MLTS program. Based on our survey results, this area's PPR vaccination participation rates (89% during and 39% outside of campaigns), seem relatively high compared to estimates of the country's overall PPR vaccination coverage of 7%. Therefore, our survey sample and results might not reflect all relevant drivers of WTV or WTP for livestock farmers throughout the country.

Another limitation is that dichotomous and polychotomous categorial variables were used with two or more categories or levels. Responses from livestock producers can be too narrow in relation to the question, such that they create or magnify bias that is not factored into the survey. For instance, on the question about satisfaction with vaccines, people might be satisfied with the intrinsic quality of vaccines but upset about the behavior of vaccinators. Combining our approach with a more quantitative approach on the household economics allowing to collect data related to household income, expenditures and budgets might help to refine further the analysis. In addition, a qualitative approach (e.g., open-ended interviews) could also help to clarify some of the producers' responses.

## Conclusion

This study focused on livestock farmers' attitudes and behaviors around vaccination, identifying socioeconomic factors that are associated with WTV animals and WTP for vaccination. These factors could be effectively managed by improving information on the benefits of vaccination, confidence in the viability of vaccines upon arrival at producers' herds, the qualifications of private veterinarians in charge of vaccination, vaccine pricing transparency, and improved information sharing about vaccination campaigns.

## Data Availability Statement

The datasets generated for this study are available on request to the corresponding author.

## Ethics Statement

This study was implemented in the framework of a larger programme that aimed at improving access of livestock producers to veterinary inputs in Mali. All participants to this specific study were already enrolled by the programme to receive interventions for increasing vaccination coverage against PPR in their areas. For the individual interviews, informed oral consents were obtained from all participants. In addition, an official approval was obtained from the National Directorate of Veterinary Services in accordance with their national mandate to carry out post-vaccination sero-monitoring for PPR and evaluate vaccination campaigns in the target regions (approval reference number N0057/MEP-DNSV).

## Author Contributions

All authors listed have made a substantial contribution to the work and approved for publication. MD, AW, AF, and BW conceived the study. AW, MD, and AY supervised the data collection and performed the preliminary data analysis. MD, BW, and AF developed and implemented the PPR control in Mali. AW and KR supervised and performed the economic analysis on WTV and WTP.

### Conflict of Interest

The authors declare that the research was conducted in the absence of any commercial or financial relationships that could be construed as a potential conflict of interest.

## Footnotes

^1^http://www.fao.org/emergencies/resources/documents/resources-detail/en/c/282777/

^2^Producers sometimes have a little trouble judging the quality of a vaccine. Some still use observations (such as the texture of the product) to get an idea of the quality and decide whether or not to vaccinate. Having a detector that can immediately show whether the vaccine is good or not could greatly help.

^3^Intuitively, it is worth bearing in mind that attributes that are to be focused on in the WTP work would come from the WTV analysis that precedes it. In this study, even though WTV is a sine qua non condition of WTP, we opted to test two key criteria related to access to vaccines and the true or false perception of the quality of vaccines.

^4^Mali is facing increased security threats and a protracted political crisis. This raises the security-risk level across the country and constraints interview-based fieldwork.
